# DNA Display I. Sequence-Encoded Routing of DNA Populations

**DOI:** 10.1371/journal.pbio.0020173

**Published:** 2004-06-22

**Authors:** David R Halpin, Pehr B Harbury

**Affiliations:** **1**Department of Biochemistry, Stanford University School of MedicineStanford, CaliforniaUnited States of America

## Abstract

Recently reported technologies for DNA-directed organic synthesis and for DNA computing rely on routing DNA populations through complex networks. The reduction of these ideas to practice has been limited by a lack of practical experimental tools. Here we describe a modular design for DNA routing genes, and routing machinery made from oligonucleotides and commercially available chromatography resins. The routing machinery partitions nanomole quantities of DNA into physically distinct subpools based on sequence. Partitioning steps can be iterated indefinitely, with worst-case yields of 85% per step. These techniques facilitate DNA-programmed chemical synthesis, and thus enable a materials biology that could revolutionize drug discovery.

## Abstract 2

Resin-linked oligonucleotides are described that efficiently partition subpools of DNA based on sequence, enabling DNA- programmed chemical synthesis

## Introduction

Subsequent to the discovery of DNA as the information-carrying blueprint for biopolymer assembly, the possibility has existed for its utilization to program molecular processes devised by man. DNA is an attractive material for several reasons. It provides very high information density: a micromolar solution of thousand-base DNA fragments can store 10^6^ bits per femtoliter. The information is amplifiable, so that a single molecule can be copied to produce a measurable quantity of nucleic acid. A large collection of enzymatic tools (e.g., polymerases, helicases, recombinases, and restriction enzymes) and man-made tools (e.g., oligonucleotide synthesizers, thermal cyclers, and purification kits) exist to manipulate DNA. Several technologies take advantage of these facts. For example, patterned DNA fragments have been used to direct self-assembly of nucleic acid objects (Seeman 2003), to follow the fate of cells in complex populations (Shoemaker et al. 1996), to localize substrates and catalysts for “lab on a chip” experiments (Winssinger et al. 2002), and for DNA computing (Braich et al. 2002). More recently, the idea has been advanced that patterned DNAs could be used to direct small-molecule synthesis (Harbury and Halpin 2000; Gartner and Liu 2001), providing a genetic code for organic chemistry.

A fundamental difficulty in using DNA to program molecular events is transducing the information contained within a nucleic acid sequence into a corresponding physical outcome. One general scheme to link DNA identity to a downstream process relies on sequence-specific partitioning. This self-separation is accomplished straightforwardly by hybridization of DNA molecules to immobilized oligonucleotides. Once spatially separated, the different pools of nucleic acid can be subjected to different processing steps. Thus, the sequence of a DNA fragment determines its fate. For multistep procedures, sequential hybridizations to multiple subsequences within a DNA molecule are required. Iterative partitioning of DNA molecules is equivalent to routing the molecules through a network, with each sequence taking a unique path.

Routing small quantities of DNA requires high-yielding, high-fidelity, and repeatable preparative hybridization. Although a vast literature exists on DNA hybridization for analytical purposes, literature on preparative applications, where DNA must be recovered after hybridization, is quite limited. Major precedents include RNA purification over polyrA binding resins (Aviv and Leder 1972) or tRNA binding resins (Tsurui et al. 1994), and an electrophoresis-based selection procedure used in DNA computing (Kenney et al. 1998). However, none of these methods is suitable for routing, either because they are not efficient, not repeatable, or difficult to interface with a downstream physical outcome. Here we present a practical method to autoroute DNA libraries through multiple decision points of a tree-type network, making DNA-programmed assembly processes possible.

## Results

**Figure 1:**
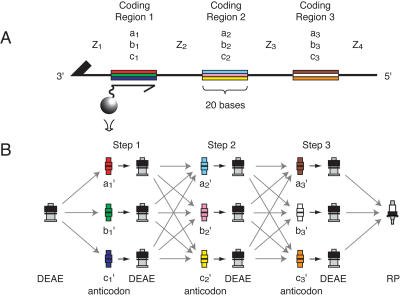
Routing DNA through Networks (A) Structure of a simplified nine-member routing gene library. The ssDNA consists of 20-base noncoding regions (black lines Z_1_–Z_4_) and 20-base coding positions (colored bars [a,b,c]_1–3_). All library members contain the same four DNA sequences at the four noncoding regions. At each of the three coding positions, three mutually exclusive codons, (a,b,c)*_n_*, are present for a total of twenty-seven different routing genes. Resin beads coated with an oligonucleotide complementary to one codon (anticodon beads; gray ball at left) capture by hybridization ssDNAs containing the corresponding codon. (B) To travel through the network, the ssDNA library starts on one or multiple DEAE columns (black column on left) and is hybridized to a set of anticodon columns (red, green, and blue columns) corresponding to the set of codons in the first coding position. The genes are thus physically partitioned into subpools based on sequence identity and can be processed accordingly. Each subpool is subsequently transferred to a distinct DEAE column, completing the first step through the network. The hybridization splitting, processing, and transfer are repeated for all subsequent coding regions. After completion of the final step, the library is concentrated on a reverse-phase column (RP; black-and-white column on right) and eluted for solution manipulation.

Our experimental scheme is illustrated in Figure 1. A population of DNA “genes” consisting of catenated coding positions is constructed. Defined sets of “codon” sequences exist at the first position (a_1_, b_1_, and so on), second position (a_2_, b_2_, and so on), and subsequent positions. Codons present in one coding position are mutually exclusive of the codons at any other position. The identity of the first codon determines the fate of the gene at the first decision point of the network, the second codon at the second decision point, and so forth.

Genes are “read” by hybridization to a set of anticodon columns. Each anticodon column displays an oligonucleotide complementary to one codon sequence, and a complete set of columns comprises the complements to all codons at a single coding position. Genes bind to the columns by codon–anticodon base pairing, and are thus partitioned. To read a subsequent coding position, the genes are first transferred to a nonspecific DNA binding resin, regenerating an unhy-bridized state. This DNA is then hybridized to a new set of anticodon columns. By a series of such reading cycles, the sequence of a gene guides it through the network.

### DNA Routing Genes

For DNA routing genes, we adopted a modular design adaptable to networks of varying depth and width. We chose codons consisting of 20 bases, catenated to neighboring codons through 20-base noncoding regions (Figure 1). To prevent aberrant codon–anticodon pairing, all sequences were taken from a set of more than 10,000 distinct 20mers that do not crosshybridize in microarray experiments (Giaever et al. 2002). The work reported here utilized 340-base fragments that specified routes through a tree with eight hierarchical levels and ten branches per level. Each of the 10^8^ unique genes contained routing instructions for eight decision points.

**Figure 2:**
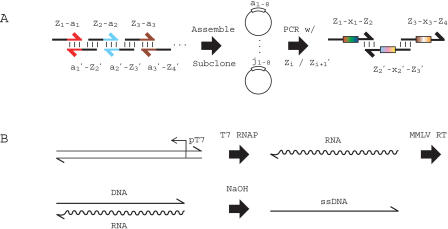
Construction and Diversification of Routing Gene Populations (A) Overlapping complementary oligonucleotides that span an entire gene (for example [Z–a]_1–8_ and a_1–8_′–Z_2–9_′) were assembled into full gene products (“all a,” “all b,” etc.) by primerless PCR and subcloned. Equivalent amounts of the ten resulting plasmids (a_1–8_, … , j_1–8_) were mixed and used as template for eight separate PCR reactions with noncoding region primer pairs (Z*_i_*/Z*_i_*_+1_′) that flanked a single coding position. The eight degenerate PCR products (Z*_n_−x_n_−*Z*_n_*_+1_) were assembled into a library of 10^8^ different genes by primerless PCR (right). (B) To generate ssDNA, a T7 promoter (pT7) was appended to the 3′ end of the double strand DNA library. The minus strand of the library was transcribed using T7 RNA Polymerase (T7 RNAP), and reverse transcribed from a Z_1_ primer using MMLV Reverse Transcriptase (MMLV RT) in a coupled reaction. The resulting DNA/RNA heteroduplex was treated with sodium hydroxide to hydrolyze the RNA, providing ssDNA.

Construction of the gene library proceeded in two stages (Figure 2A). Initially, 160 40-base oligonucleotides comprising a codon and an adjacent noncoding region were synthesized. We assembled sets of 16 of these 40mers (for example, the oligonucleotides corresponding to codons a_1_, a_2_, . . . , a_8_) into ten 340-base genes (“all a,” “all b,” etc.). The ten different genes were subcloned. Eight 60-base segments were then PCR amplified from an equimolar mixture of the parental plasmids. Each segment consisted of a coding position and two adjacent noncoding regions. The eight degenerate products were spliced together into 340-base fragments by primerless PCR, thus producing a library of 10^8^ complexity. In principle, collective assembly of the 160 40mer oligonucleotides would have created the library in one step. In practice, the two-stage approach provided better control over codon distributions in the final gene population.

The noncoding regions play an instrumental role in the construction and handling of genes. By providing conserved crossover points, they facilitate the modular generation of highly complex populations from a small number of starting oligonucleotides. For the same reason, the noncoding regions make it possible to diversify existing gene sets by recombination. The noncoding regions also place codons in the correct coding position, ensuring that all genes incorporate one codon per branch point of a network. The existence of a well-defined “reading frame” results from the fact that anticodon columns only hybridize to DNA subsequences at a specified coding position, and not to codons elsewhere in the gene.

To obtain hybridization-capable nucleic acid, the duplex DNA genes must be converted to a single-stranded form. (Possibly, duplex DNA hybridization to oligonucleotides through D-loops could be driven by RecA and ATP [Shortle et al. 1980]). A number of approaches for generating single-stranded DNA (ssDNA) have been described (for example Nikiforov et al. 1994; Williams and Bartel 1995; Pagratis 1996; Ball and Curran 1997), but we found most of them unsuitable for large-scale work. A modified nucleic-acid-sequence-based amplification protocol (Compton 1991) ultimately proved most expedient. Thus, we appended a T7 polymerase promoter to duplex DNA routing genes by PCR amplification with appropriate primers (Figure 2B). This material was used as the substrate for a coupled transcription/reverse-transcription reaction to generate DNA/RNA heteroduplexes. Hydrolysis of the RNA strand of the heteroduplexes with sodium hydroxide provided nanomole quantities of high-quality single-stranded DNA.

### Oligonucleotide Hybridization Chromatography

Synthesis of anticodon columns involves covalent attachment of oligonucleotides to a solid phase. Thiol-containing and amine-containing oligonucleotide modification reagents are commercially available, and either should facilitate coupling to an appropriately activated resin. However, pilot experiments indicated that amide linkages were more easily prepared than thioether linkages. The deprotection protocols for oligonucleotide-linked sulfhydryl moieties were more complex than for amine moieties, and sulfhydryl-modified oligonucleotides were prone to oxidation and general loss during manipulation steps. Amine-modified oligonucleotides were easier to work with and were thus used for production of anticodon columns. It proved necessary to desalt crude oligonucleotides over reverse-phase cartridges before coupling.

**Figure 3:**
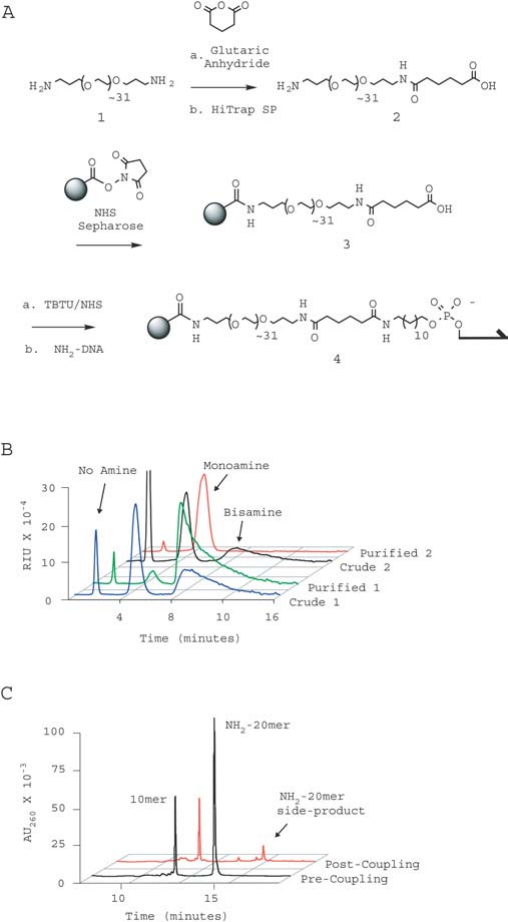
Anticodon Column Synthesis (A) Jeffamine 1500 (compound 1) was reacted with glutaric anhydride, and the singly acylated linker (compound 2) was purified over a HiTrap SP column. Purified compound 2 was coupled to NHS-activated Sepharose (gray ball). Treatment of the linkered resin compound 3 with TBTU/NHS, and subsequent incubation with a 5′-amino modified oligonucleotide (NH_2_-DNA), completed the synthesis of an anticodon column. (B) Refractive index FPLC chromatograms of PEG compounds 1 and 2 before and after purification by cation-exchange chromatography. Linker compound 1 migrates as a bisamine (green trace) while compound 2 migrates as a monoamine (red trace). (C) HPLC chromatograms of a 5′-aminated 20-base oligonucleotide (NH_2_-20mer) and a nonaminated ten-base oligonucleotide control (10mer) incubated with TBTU/NHS activated resin compound 3. Chromatograms of the starting material (black) and supernatant after 12 h (red) are shown. An unknown side-product of the coupling reaction (NH_2_-20mer side-product) is labeled.

As candidate solid phases, we tested commercially available chromatography resins made of polystyrene (Magnapore macroporous chloromethylpolystyrene beads, Argogel-NH2, epoxide-activated Poros 50 OH), methacrylate (Ultralink Biosupport Mediumand Iodoacetyl), and agarose (*N*-hydroxysuccinimide[NHS]-activated Sepharose, carbonyl diimidazole-activated Sephacryl S-1000). 20-base modified anticodon oligonucleotides were coupled to the resins. Quantification by reverse-phase chromatography of the uncoupled oligonucleotide remaining in solution provided a measure of reaction progress (Figure 3). An underivatized ten-base oligonucleotide was included in all coupling reactions to control for nonspecific loss of nucleic acid.

**Figure 4:**
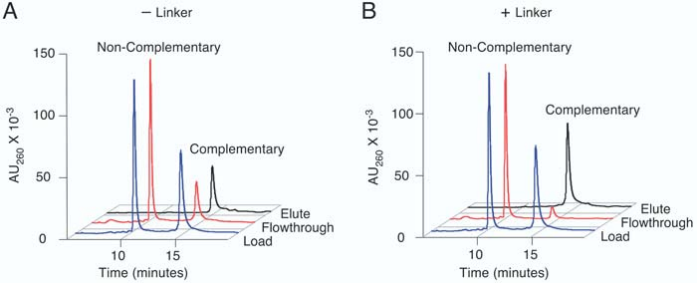
Linker Effects on Hybridization The hybridization to anticodon columns of a ten-base noncomplementary oligonucleotide and a 20-base complementary oligonucleotide was analyzed by HPLC. Chromatograms of the hybridization load (blue), flow-through (red), and elute (black) are shown. (A) shows the anticodon column that was synthesized by coupling the anticodon oligonucleotide directly to NHS-activated Sepharose. (B) shows the anticodon column that was synthesized by coupling the anticodon oligonucleotide to NHS-activated Sepharose through a PEG linker.

To test hybridization properties, 50 μl columns of the derivatized resins were loaded with 1 nmol each of a complementary 20-base oligonucleotide and a noncomplementary ten-base oligonucleotide by cyclical flow in high-salt buffer. After column washing, bound oligonucleotides were eluted with deionized water. The specificity and efficiency of hybridization were evaluated by high performance liquid chromatography (HPLC) analysis of the load, flow-through, and elute fractions (Figure 4). By this assay, none of the initial resins functioned for preparative DNA fractionation, either because they failed to bind DNA well (Argogel-NH2, epoxide-activated Poros 50 OH, NHS-activated Sepharose, and Biosupport Medium) or because they bound DNA without sequence specificity (Magnapore beads and Iodoacetyl).

Following an observation that long polyethylene glycol (PEG) linkers dramatically improve hybridization to DNA on polypropylene surfaces (Shchepinov et al. 1997), we synthesized an approximately 100-atom modified PEG spacer to sit between the resin and the anticodon oligonucleotide (see Figure 3). The synthetic scheme utilized inexpensive, commercially available reagents and ion-exchange chromatography for purification. Efforts to attach the spacer to Biosupport Medium were unsuccessful, but the spacer coupled readily to NHS-activated Sepharose and to carbonyl diimidazole-activated Sephacryl S-1000. The linkered Sephacryl and Sepharose materials immobilized amine-modified oligonucleotides to final densities of approximately 90 nmol per milliliter of resin (Figure 3C). Anticodon columns containing 50 μl of either resin efficiently and reversibly hybridized to 1 nmol of a complementary 20-base oligonucleotide, while exhibiting unmeasurable binding to a noncomplementary ten-base oligonucleotide (Figure 4). Subsequent experiments were carried out with the Sepharose-based resin. The hybridization columns proved extremely robust, withstanding over 30 hybridization cycles, treatment with 10 mM sodium hydroxide, and exposure to dimethylformamide (DMF) without a detectable decrease in perfor-mance.

### Fidelity of Routing

**Figure 5:**
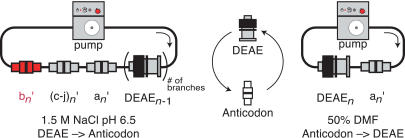
Cyclical Multistep Routing (Left) Genes are transferred from the “*n−*1” step DEAE columns to the “*n*” step anticodon columns by connecting all columns in series and cyclically pumping a high-salt buffer through the system with a peristaltic pump (gray box) for 1 h at 70 °C and 1 h at 46 °C. (Right) Genes are transferred from an “*n*” step anticodon column to an “*n*” step DEAE column by connecting the two columns in series and cyclically pumping 50% DMF through the system for 1 h at 45 °C. Arrows indicate the direction of flow.

We next investigated how buffer conditions and temperature influenced the accuracy and yield of 340-base ssDNA hybridization to anticodon columns. For these experiments, a single radiolabeled DNA gene was diluted 10-fold into an excess (50 pmol) of an unlabeled routing gene library, and loaded onto a 250-μl diethylaminoethyl (DEAE) Sepharose column. The DEAE Sepharose column was placed in a closed 3-ml fluid circuit containing ten anticodon columns, of which only one complemented a codon within the radiolabeled DNA. Hybridization buffer was pumped over the system in a direction that placed the complementary anticodon column distal to the DEAE Sepharose column (Figure 5, left). After hybridization, the flow-through was collected, and bound nucleic acid was eluted off of each column in the system. The quantity of radiolabeled DNA present in each fraction was determined by scintillation counting.

By varying the temperature (25 °C to 70 °C), salt identity (sodium chloride, lithium chloride, or tetramethylammonium chloride) and salt concentration (10 mM to 2 M) of the hybridization buffer, we determined that 1.5 M sodium chloride in a phosphate buffered solution with pH 6.5 at 45 °C provided the most robust hybridization behavior over multiple codon sequences. In addition, an initial high temperature step (70 °C) and the presence of a DEAE column inline proved critical to achieving uniformly high yields (Table 1). The high temperature and DEAE column may serve to break up structures in the DNA genes that inhibit association with anticodon columns. Consistent with previous microarray data (Shoemaker et al. 1996), addition of 20-base oligonucleotides complementary to the noncoding regions improved hybridization efficiency. The hybridization kinetics were fast, approaching equilibrium to within 5% in less than an hour at 46 °C. Using optimal hybridization conditions, 90% or more of the input radiolabeled DNA was routed to the correct anticodon column irrespective of the sequence pair used.

**Table 1:**
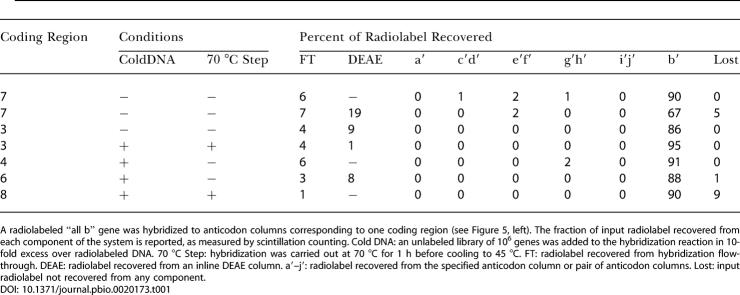
340-Base ssDNA Hybridization Efficiencies and Specificities

A radiolabeled “all b” gene was hybridized to anticodon columns corresponding to one coding region (see Figure 5, left). The fraction of input radiolabel recovered from each component of the system is reported, as measured by scintillation counting. Cold DNA: an unlabeled library of 10^6^ genes was added to the hybridization reaction in 10-fold excess over radiolabeled DNA. 70 °C Step: hybridization was carried out at 70 °C for 1 h before cooling to 45 °C. FT: radiolabel recovered from hybridization flow-through. DEAE: radiolabel recovered from an inline DEAE column. a′–j′: radiolabel recovered from the specified anticodon column or pair of anticodon columns. Lost: input radiolabel not recovered from any component

### Serial Multistep Routing

In order to route a DNA fragment through successive levels of a hierarchical tree, multiple hybridization steps are required. DNA from parental anticodon columns must be isolated and hybridized to anticodon columns corresponding to the daughter-node branches. The manipulations must be highly efficient to ensure good routing yields through trees with many levels.

We investigated several schemes for accomplishing iterative hybridizations. Our initial strategies utilized a multi-step procedure with three columns (anticodon, DEAE, and reverse-phase), linear transfer formats, and centrifugal evaporation. These three-column strategies did not prove to be high-yielding. We eventually observed that efficient iterative hybridizations could be accomplished with only two columns, using cyclic column-to-column transfers. Thus, a parental anticodon column with bound DNA was placed in a liquid circuit with a 250-μl DEAE-Sepharose column (Figure 5, right). A 50% DMF solution was pumped over the system, breaking interactions between the anticodon column and bound DNA, and promoting the binding of free DNA to the anion-exchange resin. (DNA bound to DEAE columns can be conveniently interfaced with an encoded process, such as covalent transformation by solid-phase organic chemistry [Halpin et al. 2004]). Subsequently, the DEAE-Sepharose column with bound DNA was placed in a liquid circuit with a set of anticodon columns corresponding to the branches of the daughter node. As described above, a high-salt buffer was pumped cyclically over the system to elute DNA from the anion-exchange resin, and to promote hybridization to the new set of anticodon columns. The two reciprocal transfers constitute one hybridization cycle and can be repeated indefinitely. The iterative hybridizations proceed with very high DNA recoveries (greater than 95% for anticodon to DEAE and greater than 90% for DEAE to anticodon) for several reasons. First, the columns “see” a large volume of liquid flow in the cyclic format, although the total volume of buffers used is small. Second, because the transfers are column-to-column, losses associated with manipulation of dilute DNA solutions do not occur. The two-column strategy makes it practical to iterate successive hybridizations, with worst-case overall yields of 0.85*^n^* for *n* hybridizations.

The final requirement was to isolate DNA as a concentrated, salt-free aqueous solution upon completion of routing. For this purpose, DNA bound to anticodon columns was eluted with a small volume of an (ethylenedinitrilo)tetraacetic acid (EDTA) solution and precipitated. Alternatively, DNA bound to DEAE-Sepharose columns was transferred to a reverse-phase cartridge by cyclically pumping a high-salt buffer over the two columns in series. DNA on the reverse-phase cartridge was then washed with deionized water, eluted in acetonitrile/water, concentrated by evaporation, and desalted over a microcentrifuge gel-filtration column.

## Discussion

Several technical improvements to our protocols are possible. The hybridization conditions could be further optimized to increase yields and shorten times, perhaps by the addition of proteins such as Escherichia coli SSB or RecA (Nielson and Mathur 1995). For procedures involving multiple generations of a gene population, a T7 promoter must be appended repeatedly to the library. Incorporation of an RNAZ module into the routing genes would eliminate this step by providing a permanent T7 promoter (Breaker et al. 1994). Conceivably, ssDNA production could be rendered unnecessary by using peptide nucleic acids as capture oligonucleotides or as complements to the noncoding regions. Peptide nucleic acids have been reported to invade DNA duplexes, forming more stable heteroduplexes (Kuhn et al. 2002).

Routing of DNA populations provides a general way to exploit DNA as a programming medium. For example, a routing approach has been utilized to compute solutions to the traveling salesman problem (Braich et al. 2002). In order to obtain the answer, it was necessary to isolate DNA fragments containing a defined set of subsequences through iterated hybridizations. By increasing the speed and yield of such isolation steps, the tools described here should aid DNA computing advances.

The preparative hybridization protocols will also facilitate purification of defined genomic sequences and primary mRNA transcripts for the study of nucleic acid modifications, and for the analysis of adjunct proteins. One advantage of iterated hybridization in this context is that it increases the overall specificity of purification, in much the same way that successive amplifications with nested primers increase the specificity of PCR reactions. The technique could also be applied to the isolation of nucleoprotein complexes, such as telomerase, that have been tagged with nucleic acid epitopes.

Finally, the fates of individual molecules undergoing a process of covalent assembly can be programmed by routing. For example, the protocols presented here were used to direct the split-and-pool synthesis of a combinatorial chemistry library (Halpin and Harbury 2004). That work involved routing genes through a tree with six levels and ten branches per level. In order to program large libraries of very low-molecular-weight compounds, routing through shallow trees with thousands of branches per level will be required. Adaptation of the anticodon columns to a microarray format would achieve this goal in a practical manner. Such massively parallel DNA-directed chemistry has the potential to revolutionize modern drug discovery.

## Materials and Methods

### 

#### Materials.

*O*,*O′*-bis(3-aminopropyl) polyethylene glycol of average molecular weight 1500 Da (compound #14535-F, also called Jeffamine 1500) and all other chemicals and solvents were purchased from Sigma-Aldrich (St. Louis, Missouri, United States). DEAE columns were prepared by pipetting approximately 250 μl of DEAE Sepharose Fast Flow resin (#17-0709-01; Amersham Biosciences, Little Chalfont, United Kingdom) into empty TWIST column housings (#20-0030; Glen Research, Sterling, Virginia, United States).

#### DNA library assembly.

160 40mer oligonucleotides were synthesized using standard phosphoramidite chemistry. The 5′ 20 bases of each oligonucleotide consisted of a noncoding region sequence, while the 3′ 20 bases consisted of a codon sequence. Oligonucleotides were purified by electrophoresis on 15% denaturing acrylamide gels. The oligonucleotides were divided into ten sets exclusive to a-type codons (a_1_–a_8_), b-type codons (b_1_–b_8_), and so forth. Each set of 16 oligonucleotides was assembled into a 340-base fragment by primerless PCR (Stemmer et al. 1995). Assembly reactions contained 1 μl Vent DNA Polymerase (#0254; New England Biolabs, Beverly, Massachusetts, United States), 1X Vent buffer, 250 μM each dNTP, and 0.1–10 pmol of each oligonucleotide, and were run for 20 to 35 cycles (unless otherwise noted, PCR reactions had a volume of 100 μl). In a second PCR step, the assembly products were amplified from 1 μl of the assembly reaction using 0.2 nmol of each end primer. The amplified genes were purified on 2% NuSieve (#50081; FMC BioProducts, Rockland, Maryland, United States) agarose gels and subcloned between the SphI and EcoRI sites of the pET24A plasmid (#70769-3; Novagen, Madison, Wisconsin, United States). To construct a full library, the ten plasmids were mixed in equal proportions and used as template for eight PCR reactions. The primers were Z_1_/Z_2_′ for the first reaction, Z_2_/Z_3_′ for the second reaction, and so forth. The resulting 60-base-pair products were purified on 3% NuSieve agarose gels. Following quantification by densitometry, 120 ng of each fragment was used in a single 50-μl, ten-cycle primerless PCR reaction to assemble a library. The assembly products were amplified using 1 μl of the assembly reaction as template and 0.2 nmol of each end primer. The final library was subcloned, and 36 isolates were sequenced to verify the presence of the expected codon distribution at each coding position.

#### Preparation of ssDNA.

ssDNA was generated using a modified NASBA reaction (Compton 1991). Duplex DNA template (1–10 pmol) was transcribed/reverse-transcribed in a 200-μl reaction containing 1 nmol primer, 40 mM Tris (pH 8.3), 20 mM magnesium dichloride, 40 mM potassium chloride, 10% DMSO, 5 mM DTT, 0.1 mg/ml BSA, 3.5 mM each rNTP, 2.5 mM each dNTP, 1000 U MMLV RNAseH minus reverse transcriptase (for example #M3682; Promega, Madison, Wisconsin, United States), 100 U T7 RNA Polymerase (for example Promega #P2075), and 2 U of pyrophosphatase (New England Biolabs #MO296). To prepare radiolabeled ssDNA, a primer kinased with γ-^33^P ATP was used. Reactions were incubated for 12 h at 42 °C. Following the enzymatic step, RNA was hydrolyzed by addition of sodium hydroxide to 100 mM and heating of the reaction tube for 2 min at 100 °C. The solution was subsequently neutralized with acetic acid and spun in a benchtop microfuge at 16,000 g for 2 min to remove precipitated material. The supernatant was transferred to a fresh tube, brought to 50 mM EDTA, and ethanol precipitated. ssDNA product was purified by electrophoresis on 4% denaturing acrylamide gels. Excised gel bands were crushed and rotated overnight in 3–6 ml 5 mM Tris (pH 8.0), 500 μM EDTA, and 500 μM EGTA. Acrylamide was removed by spin column filtration, and the solution volume was reduced to 800 μl by centrifugal evaporation. Samples were phenol/chlorofom extracted, ethanol precipitated, and resuspended in water.

#### Purification of bisamine linker (compound 1).

The crude Jeffamine material was purified by fast protein liquid chromatography cation-exchange chromatography over a 5-ml Hi-Trap SP column (Amersham Biosciences #17-1152-01). In early work, 1-ml batches of a 250-mg/ml aqueous solution were loaded onto the column in 50 mM acetic acid, washed with load buffer, and bumped off with 1 M lithium chloride. Subsequently, we developed a gradient protocol. The material was loaded in water, and the product was eluted with a linear water–hydrogen-chloride gradient (0–30 mM hydrogen chlo-ride over 15 column volumes) at 6 °C monitored by refractive index detection (RID-10A; Shimadzu, Tokyo, Japan). After every fifth injection, the column was washed with 1.5 M sodium chloride to remove a yellow residue and was then reequilibrated in deionized water. Pooled fractions of the bisamine peak were brought to pH 10 by addition of solid sodium carbonate, and the purified Jeffamine product (compound 1) was extracted into methylene chloride. The combined organic layers were dried over sodium sulfate, and solvent was removed by rotary evaporation. Yields of the pale yellow solid were 40% based on the weight of crude starting material.

#### Synthesis of amine-acid linker (compound 2)

One mole equivalent of 1.5 M glutaric anhydride in dioxane was added to a briskly stirred 250-mg/ml aqueous solution of purified Jeffamine (compound 1). After 30 min, the crude reaction product was injected in 1-ml batches over a 5-ml Hi-Trap SP column and eluted as described above. Pooled fractions of the monoamine peak were brought to pH 7 by addition of solid sodium bicarbonate, and the purified linker product (compound 2) was extracted into methylene chloride, dried over sodium sulfate, and obtained as a pale yellow solid by rotary evaporation. Yields were 35% to 50% based on the weight of purified Jeffamine starting material. A compound similar to the purified linker compound 2 is commercially available (#0Z2W0F02; Nektar Therapeutics, San Carlos, California, United States).

#### Synthesis of linkered resin (compound 3).

To prepare resin compound 3, compound 1 or compound 2 was dissolved at 300 mg/ml in DMF/200 mM DIEA. The linker solution was incubated with one volume equivalent of drained NHS-activated Sepharose (Amersham Biosciences #17-0906-01). The suspension was rotated at 37 °C for 72 h, washed over a plastic frit with DMF to remove excess linker, and incubated with 1 M ethanolamine in DMF for an additional 12 h at 37 °C. The product resin was washed and stored at 6 °C. Resins coupled to compound 1 were further treated by incubation with an equal volume of DMF containing 100 mM glutaric anhydride and 15 mM pyridine at 37 °C under rotation for 48 h.

#### Resin activation (compound 4).

Typically, TBTU (320 mg) and NHS (115 mg) were dissolved in 4 ml of DMF/500 mM DIEA, and drained compound 3 (1 ml) was added. The suspension was rotated at 37 °C for 1 h. Product resin was washed with the following sequence (20 ml of each): ethyl acetate, tetrahydrofuran, ethanol, water, 5 M sodium chloride, water, and DMF. Resin activation was performed just prior to oligonucleotide coupling.

#### Construction of anticodon columns

Twenty-base capture oligonucleotides were synthesized using standard phosphoramidite chemistry, with the addition of a C12-methoxytritylamine modifier at the 5′-end (Glen Research #10-1912). Following ammonia cleavage and drying, the oligonucleotides were desalted over C18 Sep-Pak cartridges (#WAT020515; Waters Corporation, Milford, Massachusetts, United States). Purification proceeded according to the manufacturer's instructions, but a deionized water wash was inserted before the final elution step to remove residual TEAA. Coupling reactions of oligonucleotides to resin were carried out in low-binding 0.65-ml microcentrifuge tubes (#11300; Sorenson Bioscience, Salt Lake City, Utah, United States). Ten nanomoles of a capture oligonucleotide and 10 nmol of a nonaminated 10mer control oligonucleotide in a 40-μl aqueous solution were mixed with 160 μl of DMF/200 mM DIEA. Fifty microliters of drained resin compound 4 was added, and the suspension was rotated at 37 °C for 12 h. Reaction progress was monitored by HPLC. Supernatant aliquots (20 μl) were injected onto a 4.6-mm × 25-cm Varian Microsorb-MV 300-5 C18 column (#R0086203C5; Varian, Palo Alto, California, United States) and eluted with a linear water–acetonitrile gradient (0%–45% acetonitrile in five column volumes) in the presence of 0.1 M TEAA (pH 5.2) at 50 °C. After 12 h, the resin was pelleted by centrifugation at 100 g for 1 min, supernatant was removed, and the resin was incubated with 1 M ethanolamine in DMF for an additional 12 h at 37 °C. The derivatized resins were loaded into empty DNA synthesis column housings (#CL-1502-1; Biosearch Technologies, Novato, California, United States).

#### Oligonucleotide hybridization.

Hybridization was performed in a closed system consisting of an anticodon column, male tapered luer couplers (Biosearch Technologies #CL-1504-1), capillary tubing (Amersham Biosciences #19-7477-01), silicone tubing (#8060-0020; Nalgene Labware, Rochester, New York, United States), tubing connectors (Amersham Biosciences #19-2150-01, #18-1003-68, and #18-1027-62), and a peristaltic pump (Amersham Biosciences #18-1110-91). Approximately 1 ml of hybridization buffer (60 mM sodium phosphate (pH 6.5), 1.5 M sodium chloride, 10 mM EDTA, and 0.005% Triton X-100) containing 400 pmol of a complementary 20-base oligonucleotide and 400 pmol of a noncomplementary ten-base oligonucleotide was cyclically pumped through the system at 0.5 ml/min for 1 h in a 46-°C water bath. DNA was eluted off the anticodon column with 4 ml of 1 mM EDTA (pH 8.0) and 0.005% Triton X-100 heated to 80 °C. Flow-through and elute fractions were analyzed by HPLC as described above (0%–18% acetonitrile in five column volumes).

#### ssDNA hybridization.

ssDNA was loaded onto a DEAE-Sepharose column as described (Halpin et al. 2004). Anticodon columns were connected in series to the DEAE column using male tapered luer couplers, capillary tubing, silicone tubing, and tubing connectors. Approximately 3 ml of hybridization buffer containing 1 nmol of each oligonucleotide complementary to the noncoding regions was cyclically pumped over the system at 0.5 ml/min for 1 h at 70 °C, 10 min at 37 °C, and 1 h in a 46-°C water bath within a 37-°C room. Hybridized DNA was transferred back to a fresh DEAE column, or eluted with 4 ml of 1 mM EDTA (pH 8.0) and 0.005% Triton X-100 heated to 80 °C. For analysis purposes, the hybridization flow-through, the DEAE resin, and the anticodon column elutes were mixed with 10 ml of scintillation cocktail (Bio-safe 2, Research Products International, Mount Prospect, Illinois, United States) and shaken vigorously. Counting was performed using the ^35^S preset channel of a scintillation counter (Beckman Instruments, Fullerton, California, United States).

#### Anticodon to DEAE DNA transfer.

DEAE and anticodon columns were connected in series using male tapered luer couplers, 3.16-mm manifold tubing (#39-628; Rainin, Oakland, California, United States), and tygon tubing 3603. Using a peristaltic pump (Minpuls2; Gilson, Middleton, Wisconsin, United States), approximately 7 ml of a 50% DMF solution was flowed cyclically over the columns at 3 ml/min for either 1 h at 45 °C or 12 h at 25 °C.

#### Endpoint isolation of DNA.

DEAE columns were connected in series to C8 SepPak columns (Waters #WAT036775) using male tapered luer couplers, tygon tubing 3603, and tubing connectors. Approximately 6 ml of 50 mM ethanolamine (pH 10.0), 1.5 M sodium chloride, 1 mM EDTA, and 0.005% Triton X-100 was cyclically pumped over the columns at 1 ml/min for 1 h at 50 °C. The Sep-Pak columns were then washed with 12 ml of 100 mM TEAA (pH 6.5) followed by 12 ml of water. ssDNA was eluted from the Sep-Pak column with 4 ml of 50% acetonitrile heated to 80 °C. Samples were concentrated by centrifugal evaporation to a volume of approximately 30 μl and desalted over G25 Sephadex spin columns (Sigma-Aldrich #G-25-150).

## Abbreviations

DEAE: Diethylaminoethyl
DMF: dimethylformamide
EDTA: (ethylenedinitrilo)tetraacetic acid
HPLC: high performance liquid chromatography
NHS: *N*-hydroxysuccinimide
PEG: polyethylene glycol
ssDNA: single-stranded DNA
